# Autoimmune/Inflammatory Syndrome Induced by Adjuvants (ASIA) in Breast Implants: A Case Report

**DOI:** 10.7759/cureus.88418

**Published:** 2025-07-21

**Authors:** Humberto Quiroz Diaz, Alitzel Garcia Hernandez, Ivonne Castrejon Castro

**Affiliations:** 1 General Surgery, Hospital General Regional No. 1 Instituto Mexicano del Seguro Social (IMSS), Santiago de Querétaro, MEX; 2 Plastic Surgery, Hospital de Especialidades Centro Medico Nacional SXXI, Mexico City, MEX

**Keywords:** asia, autoimmunity, breast implants, explantation, induced by adjuvants, syndrome

## Abstract

Autoimmune/inflammatory syndrome induced by adjuvant (ASIA) encompasses all conditions capable of triggering systemic inflammatory processes due to the use of adjuvant materials, including the use of breast implants in plastic surgery procedures. These conditions typically cause symptoms such as pain, redness, erythema, or capsular contracture and ultimately lead to the removal of these materials, resulting in subsequent clinical improvement. We report the case of a 32-year-old woman with a history of autoimmune disease (Evans syndrome) who underwent breast implant placement and subsequently developed clinical features consistent with ASIA, accompanied by thrombocytopenia. Both clinical symptoms and laboratory parameters improved following explantation of the implants.

## Introduction

For decades, evidence has accumulated on the development of autoimmune symptoms triggered by exposure to environmental factors that act as adjuvants in susceptible hosts [1]. These adjuvants, when introduced into the body, generate a specific immune response and result in high antibody titers against a specific pathogen. Examples include silicon, aluminum hydroxide, and selenium, among others. However, it has also been clarified that medical implants, including injectable implants, silicones, and polypropylene mesh, can act as adjuvants and trigger a similar immune response [2,3].

More than one million breast augmentation procedures using implants have been performed worldwide, and in some cases, these types of adverse effects have been reported post-implantation [4]. Implants may function as adjuvants in triggering the adaptive immune response to an antigen. The development of related complications and their severity depends on a combination of genetic predisposition, environmental factors, and the presence of comorbidities.

Autoimmune/inflammatory syndrome induced by adjuvants (ASIA) refers to an immune-mediated response triggered by exposure to foreign substances, such as adjuvants found in certain medical implants, including those made of silicone. The syndrome is characterized by a wide range of nonspecific clinical manifestations, and its diagnosis is primarily one of exclusion, requiring careful differentiation from other autoimmune or systemic conditions [2].

Typical symptoms of ASIA include localized pain, hyperhidrosis, erythema, infection, capsular contracture, myalgia, arthralgia, chronic fatigue, fever, cognitive impairment, and atypical neurological symptoms [2,5]. Thus, patients typically present with severe fatigue, insomnia, difficulty falling asleep, poor concentration, weakness, and chronic pain such as myalgia and arthralgia, most of which meet criteria for fibromyalgia [4]. They have also reported, to a lesser extent, allergies, gastrointestinal symptoms, Raynaud's phenomenon, orthostatic intolerance, postural tachycardia, and cystitis.

Prior to its recognition as a syndrome in 2011, various studies documented it. Over time, new cases of ASIA have been described, and new triggering adjuvants have also been identified. In the last decade, there has been an increase in the association between the syndrome and the use of products for aesthetic purposes, including hyaluronic acid, methacrylate, polyacrylamide, and implants, as in this case [1].

## Case presentation

A 32-year-old female with a history of autoimmune hemolytic anemia diagnosed in 2020, which progressed to Evans syndrome in 2022, was admitted for evaluation of progressive thrombocytopenia and inflammatory symptoms localized to both breasts. In February 2021, she underwent breast augmentation surgery with bilateral glandular resection using Mentor brand implants (500 cc in the right breast and 550 cc in the left breast). Her personal history included chronic migraines since childhood, insulin resistance secondary to prolonged corticosteroid use, and secondary hypogammaglobulinemia, being monitored by the immunology department. A relevant family history included a mother diagnosed with rheumatoid arthritis.

**Figure 1:**
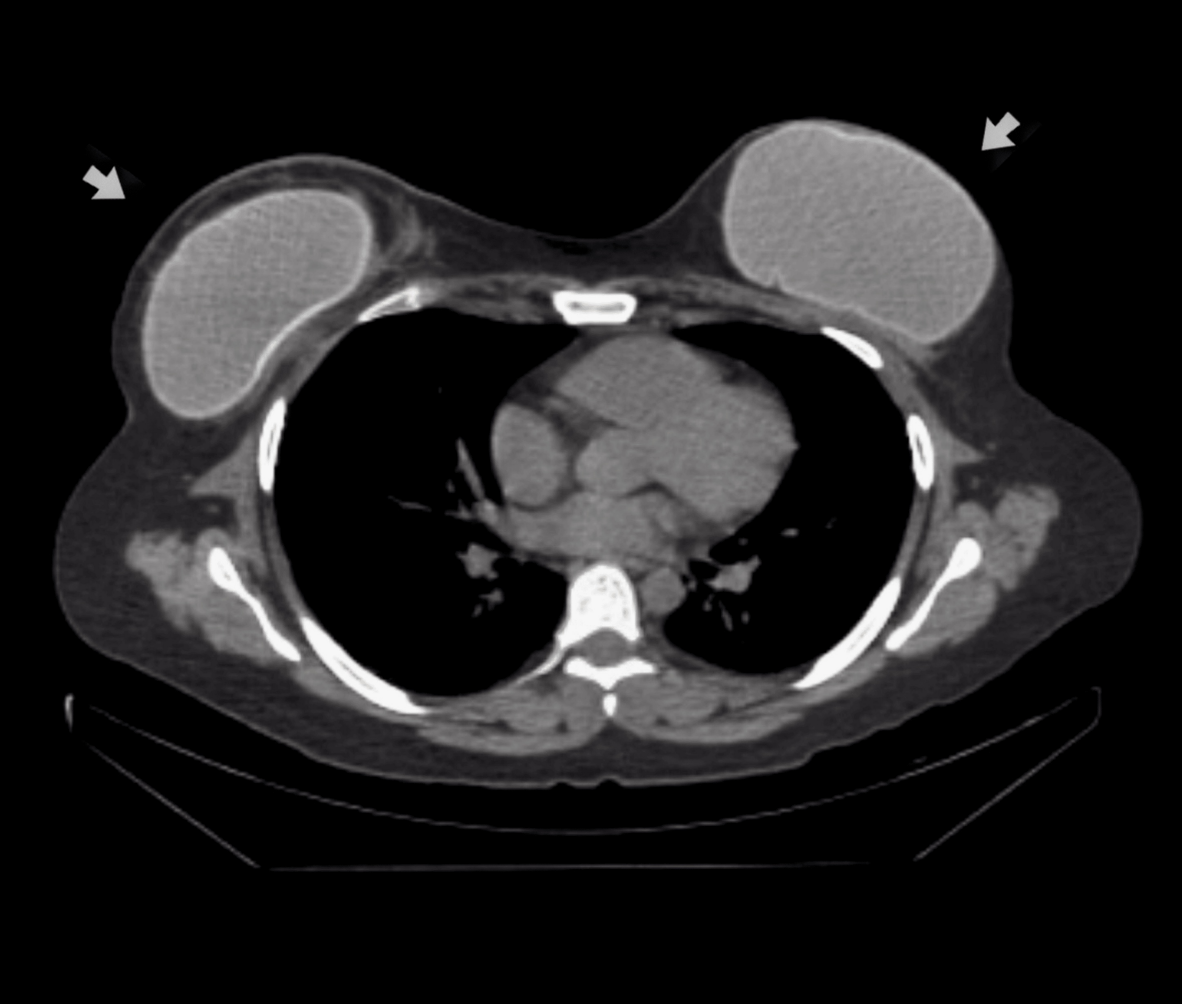
Implants that are intact in a CT: The implants are indicated by both arrows with their capsule preserved, well positioned and without signs of rupture or migration CT: computed tomography

In April 2024, the patient was admitted to the hematology department with severe and refractory thrombocytopenia (with an initial platelet count of 10,000/mm³). She also presented with symptoms in both breasts, including erythema, increased volume, pain, and localized fever in the bilateral periareolar region. Laboratory studies showed leukocytosis of 12,110/mm³ with a left shift (neutrophils 10,060/mm³) and elevated LDH (519 U/L) (Table 1). Empirical antibiotic treatment with cefepime was initiated on May 1, 2024. A simple axial CT scan taken in October 2023 demonstrates the integrity of the implants in different sections (Figure 1); however, a breast and left axillary ultrasound performed on May 9 revealed a retromammary implant with capsular retraction and findings consistent with rupture in the upper outer quadrant, with extracapsular leakage of implant material and a focal area of ​​cellulitis in adjacent soft tissues.

**Table 1:** Laboratory history from admission to postoperative period BUN: blood urea nitrogen, LDH: lactate dehydrogenase, ALP: alkaline phosphatase, GGT: gamma-glutamyl transferase, PT: prothrombin time, PTT: partial thromboplastin time, INR: international normalized ratio

Parameters	12/04/24	15/04/24	18/04/24	22/04/24	26/04/25	29/04/24	02/05/24	06/05/24	09/05/24	10/05/24	15/05/24	17/05/24	20/05/24	Normal ranges
Leukocytes (10^3^/μl)	12.1	8.7	11.5	19.7	14.2	14.0	13.8	12.2	11.7	12.4	9.2	12.5	11.8	4.6-10.2
Neutrophils (10^3^/μl)	10.0	6.9	9.3	17.7	12.3	11.3	11.3	10.1	8.55	9.44	6.6	9.45	8.6	1.5-7.0
Hemoglobin (g/dL)	13.5	12.1	11.4	12.5	13.3	13.2	11.9	11.5	12	11.2	9.0	9.7	9.6	13-18
Hematocrit (%)	41.1	38.6	34.7	38.1	40.3	40.2	36.6	35.2	37.2	34.9	28.9	30.9	31.2	42-53.6
Platelets (10^3^/μl)	10	15	13	4	7	41	29	18	39	77	49	89	280	150-450
Glucose (mg/dL)	135	92	83	114	84	98	85	75	72	76	71	78	72	70-105
Urea (mg/dL)	29.9	24.7	-	42	26	32	28	-	37	27	22	26	30	16.6-46.5
BUN (mg/dL)	14	11.5	-	19.9	12	15	13.4	-	17	12.8	10.4	12.1	14.2	6-20.6
Creatinine (mg/dL)	0.7	0.5	0.6	0.5	0.5	0.6	0.5	0.5	0.5	0.5	0.4	0.5	0.5	0.5-1.11
LDH (mg/dL) (U/L)	519	418	399	295	-	-	-		-	290	-	292	333	125-220
ALP (U/L)	16	86	-	68	75	78	-	55	58	64	55	54	61	40-150
GGT (U/L)	16	-	-	-	-	-	-	10	11	11	10	10	12	9-56
Albumin (g/dL)	4.2	3.0	-	2.8	3.0	3.1	-	2.8	3.0	2.9	2.9	3.1	3.59	3.5-5.0
Sodium (mEq/L)	135	135	-	135	-	138	139	138	137	-	138	137	138	136-145
Potassium (mEq/L)	3.3	4.0	-	3.9	-	4.1	4.8	3.8	4.4	-	4.7	4.5	4.8	3.5-5.1
Chloride (mEq/L)	103	103	-	103	-	104	-	105	105	-	105	104	103	98-107
Calcium (mg/dL)	9.3	8.5	-	8.4	-	9.5	-	-	9.4	-	8.8	-	-	8.4-10.2
PT (sec)	10.6	10.7	10.7	11.1	-	-	-	10.5	11.2	-	10.1	10.9	10.8	9.4-12.5
PTT (sec)	34.1	30	26.5	21	-	-	-	27	25.3	-	25.5	25	25.5	25.1-36.5
INR	0.98	1.0	1.0	1.04	-	-	-	0.95	1.02	-	0.94	1.02	1.01	-
D-dimer (μg/mL)	-	0.54	2,450	1,520	-	-	-	-	-	-	1.36	1.0	1.3	0.5

Based on the clinical picture and imaging findings, the plastic surgery service diagnosed grade IV capsular contracture with probable ASIA. Therefore, implant removal, capsulectomy, and possible mastopexy were indicated given the favorable preoperative conditions. The surgical procedure was performed on May 11, 2024, with bilateral explantation and partial capsulectomy. Bilateral drains were placed during the patient's hospital stay, and hematologic features, along with progressive clinical improvement, were documented in the immediate postoperative period. Intraoperative findings included an intact 500 cc left implant, purulent exudate, and an intact 450 cc right implant.

Immunomodulatory management was optimized with rituximab (once weekly, starting on April 19 for four weeks), intravenous immunoglobulin (starting on May 12), cyclosporine (starting on May 14), and a switch from deflazacort to prednisone on May 15. The antimicrobial regimen was escalated after surgery to piperacillin-tazobactam plus linezolid on May 11. During the hospitalization, progressive hematological improvement was observed, with platelet count recovering to 280,000/mm³ on May 20, 2024.

Due to the patient's exposure to prosthetic material prior to symptom onset, the presence of characteristic clinical features, rapid symptom resolution following removal of the prosthetic material, and a history of autoimmune disease, a diagnosis of ASIA associated with silicone breast implants and complicated by severe Evans syndrome was established. This diagnosis met three primary and one minor diagnostic criteria. The patient continues under multidisciplinary follow-up by hematology, immunology, and plastic surgery teams to monitor autoimmune activity and the long-term clinical course. Most departments have reduced follow-up frequency, as the patient remains clinically stable according to interdepartmental medical notes and serial laboratory findings.

## Discussion

Adverse effects associated with breast implants can be divided into subgroups such as local complications, implant migration, rupture or capsule formation, allergy to the components, or symptoms of inflammatory response, such as those seen in rheumatological diseases [1]. It is of interest to focus on the latter, as patients typically complain of fatigue, arthralgia, hair loss, hypersensitivity, and rash following implantation [1,3].

While direct evidence supporting patient susceptibility to developing ASIA after medical device implantation remains limited, several factors have been postulated, such as a history of allergies, established autoimmune diseases or familial predisposition to them, smoking, and obesity [4]. However, in contrast to rheumatological diseases, positive antibodies are not common in ASIA [1].

In general, the materials used for implants are non-immunogenic and non-toxic; however, despite these characteristics, they trigger a reaction similar to that of foreign bodies, resulting in granulomatous inflammation [1]. Patients with an allergic past medical history are at greater risk of developing ASIA after implantation. Furthermore, patients with established autoimmune disease or a familial predisposition to autoimmune disease are at risk of developing symptoms after silicone breast implantation. It's essential to consider the interplay between immunogenetic factors (i.e., human leukocyte antigens (HLA)) and environmental factors, such as smoking and obesity, in the development of medical device-induced ASIA [3]. After implantation of the material, its interaction with host cells generates the attraction of phagocytes (predominantly pro-inflammatory M1 macrophages). This process is enhanced by mast cell activation and histamine release, which also play a role in the development of pain at the implantation site secondary to the sensitization of a nociceptor V1 channel (TRPV1).

Furthermore, the implants form a microbial biofilm around them, which contributes to the chronic inflammatory response. This response is perpetuated as long as the material remains in place, acting as a danger signal to the immune system by simulating the inflammasome-activated inflammation pathway (NALP3). The NALP3 inflammasome is a primary sensing mechanism by which adjuvants, such as silicones, induce the secretion of pro-inflammatory cytokines and recruit myeloid-lineage cells [5]. As such, the implants act as adjuvants in the development of the adaptive immune response to an antigen [2].

An important criterion in ASIA analysis is to demonstrate that the symptoms that appeared with implantation of the material disappear or are reduced after its explantation. Most case series describing this improvement of the syndrome after explantation of medical devices have been published on breast implants, and improvement rates have been reported in approximately 50-98% of patients [6]. However, it is also known that not all patients benefit from explantation. Several factors have been postulated to influence the outcome of the process, including implant characteristics, disease progression, disease duration, surgical details, and whether or not post-explant reconstruction was performed.

Characteristics of implants

Breast implants can be composed of different materials, including silicone, saline, or hydrocellulose; their exterior may have a layer of silicone with smooth or textured characteristics, and they are not always 100% round. However, it has been found that capsular inflammation of the implants is more frequent in those filled with silicone compared to those filled with saline solution, which is greater on textured surfaces than in those that are smooth, and those patients who were explanted having a previous capsular contracture had a significantly better result compared to those in whom there was no capsular contracture [7,8].

Characteristics of ASIA

A study of 24,651 women with silicone implants found that those with implants had a 45% higher risk of being diagnosed with an autoimmune/rheumatologic disease compared to women without implants [7]. In patients with ASIA who already had a previous diagnosis of an autoimmune disease, post-explant recovery was not complete, and disease-modifying medications were required to further treat complications [1].

Implant duration

Increased time from implantation to explantation is inversely proportional to symptom improvement [5]. Furthermore, in another study, 59% of women who removed their implants within 10 years of placement showed significant improvement, while this figure was only 33% in those who had them for more than 10 years, confirming that, in general, exposure time plays a vital role in the progression and outcome of the syndrome [2].

Surgery

There is evidence that explantation surgery with capsulectomy confers a more pronounced improvement than in women who do not undergo it. The surgeon's experience in performing this procedure also plays a crucial role [1].

Post-explant reconstruction

After removal, reimplantation is possible for aesthetic results. This can be performed using autologous material, prosthetic material, or without reimplantation. The outcomes of patients after explantation without reconstruction, those with autologous reconstruction, and those who received a new prosthetic material were compared. Initial improvement was observed in 72% of those with reconstruction, 68% in those with a new implant, and 72% in those who received autologous reconstruction. However, symptom relapse was reported in 47% of those who received a new prosthetic implant and was uncommon in the other two groups [8].

Criteria for diagnosing ASIA

The diagnostic criteria for ASIA include both major and minor components. Primary criteria consist of exposure to prosthetic material prior to the onset of symptoms, the development of typical symptoms, significant improvement following the removal of the prosthetic material, and a normal biopsy of the involved tissues. Minor criteria include the presence of autoantibodies directed at the adjuvant material, atypical clinical manifestations such as irritable bowel syndrome or Raynaud's phenomenon, a specific HLA association, and the coexistence of another autoimmune disease. A diagnosis of ASIA is considered when two major criteria are present or when one major and two minor criteria are fulfilled.

Biochemically, elevated antinuclear antibodies have been reported in less than 20% of patients. This is the most common type of antibody, and the rarest are anti-dsDNA, anti-Scl-70, anti-polymerase III, anti-cardiolipin, anti-CCP, rheumatoid factor IgM, ANCA, or cryoglobulins. Vitamin D deficiency is common [6].

## Conclusions

With its recognition as a syndrome, new cases of ASIA due to breast implants have been studied and documented, generating a more comprehensive approach to the pathology. Women who developed ASIA after breast implants had a better outcome and improved quality of life after implant removal, and this removal confirmed the suspected diagnosis, as symptoms disappeared immediately after implant removal. As was the case with our patient, she was known to have a pre-existing autoimmune condition that was controlled. However, after implantation, she began to experience typical symptoms, meeting major and minor criteria that aided diagnosis. Subsequently, we achieved a nearly complete symptom remission rate after implant explantation. Follow-up MRI of the implants is recommended to detect capsular contracture, seroma, anaplastic lymphoma, or granulomas prior to the onset of symptoms.

Although many patients seek cosmetic surgery to enhance their appearance, it is essential to recognize the potential risks associated with such procedures, particularly in individuals with underlying autoimmune conditions. Caution should be exercised when evaluating these patients as candidates for surgeries involving adjuvant materials.
